# Spatial Transcriptomics for Tumor Heterogeneity Analysis

**DOI:** 10.3389/fgene.2022.906158

**Published:** 2022-07-05

**Authors:** Qiongyu Li, Xinya Zhang, Rongqin Ke

**Affiliations:** School of Medicine, Huaqiao University, Quanzhou, China

**Keywords:** tumor heterogeneity, spatial transcriptomics, tumor microenvironment, gene expression profiling, single-cell sequencing

## Abstract

The molecular heterogeneity of cancer is one of the major causes of drug resistance that leads to treatment failure. Thus, better understanding the heterogeneity of cancer will contribute to more precise diagnosis and improved patient outcomes. Although single-cell sequencing has become an important tool for investigating tumor heterogeneity recently, it lacks the spatial information of analyzed cells. In this regard, spatial transcriptomics holds great promise in deciphering the complex heterogeneity of cancer by providing localization-indexed gene expression information. This study reviews the applications of spatial transcriptomics in the study of tumor heterogeneity, discovery of novel spatial-dependent mechanisms, tumor immune microenvironment, and matrix microenvironment, as well as the pathological classification and prognosis of cancer. Finally, future challenges and opportunities for spatial transcriptomics technology’s applications in cancer are also discussed.

## Introduction

According to the global cancer data released by the International Agency for Research on Cancer (IARC), 19.29 million new cancer cases occurred worldwide in 2020, with a gender ratio of approximately 1.09:1 (Sung et al., 2021). At the same time, 9.96 million cancer deaths occurred worldwide, among which breast cancer ranks first in terms of incidence rate, while lung cancer ranks first in terms of mortality rate. Due to its high morbidity and mortality rates, the prevention and treatment of cancer have always been a major public concern worldwide.

**FIGURE 1:**
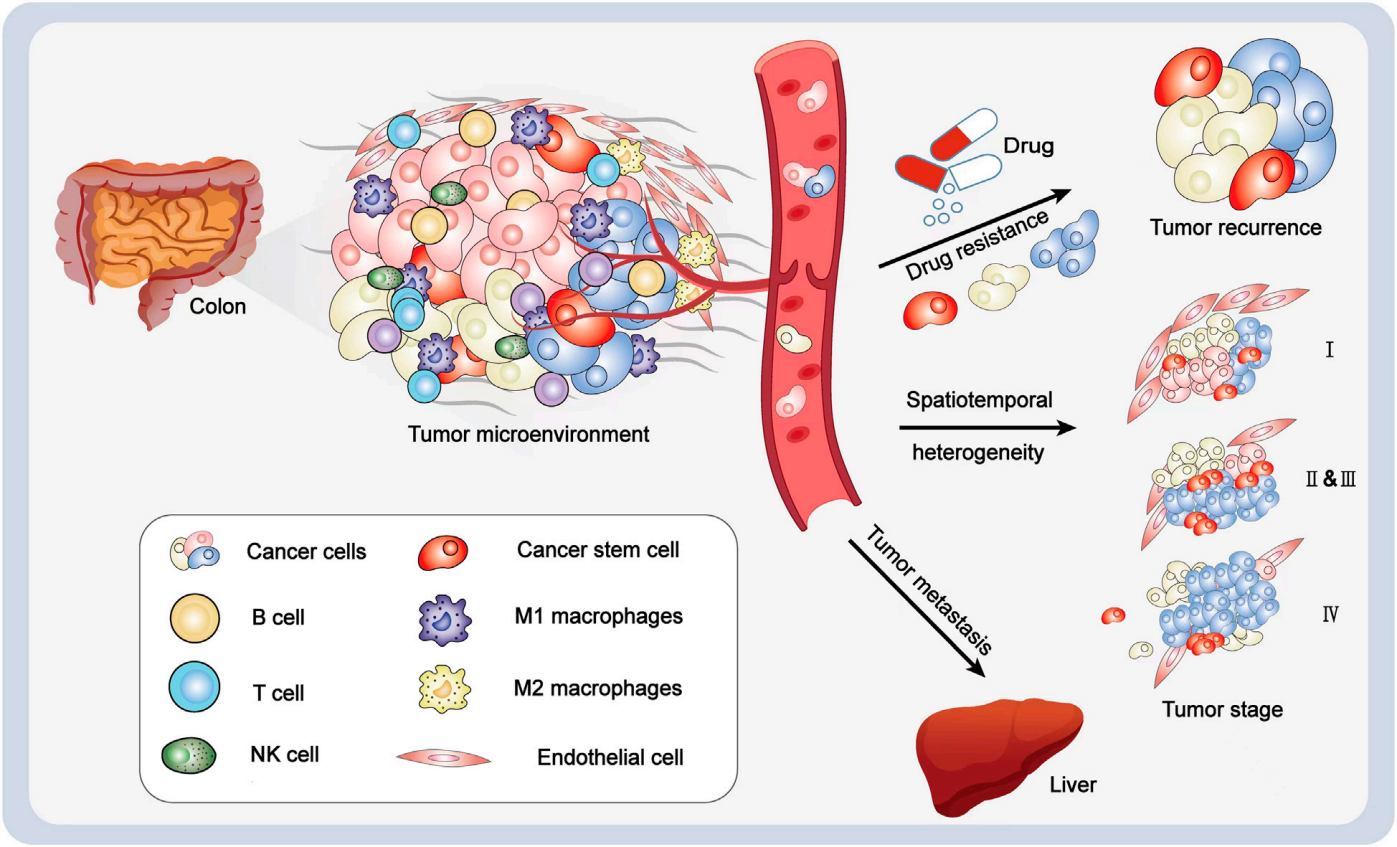
Illustration of the heterogeneity of a tumor. Cancer cells, immune cells, and stromal cells are the major components of a tumor. The interaction between microenvironment and different tumor cells makes cancer cells possess different growth potential and proliferation abilities. Cancer stem cells are the source of tumor occurrence. In the process of treatment, although most cancer cells can be eliminated through chemotherapy, drug treatment, or surgery, they can still relapse or metastasize through blood vessels and produce drug resistance. Additionally, the temporal and spatial heterogeneity of tumors will also lead to the differences in tumor treatment schemes and treatment efficacies at different times.

Malignant tumor is characterized by considerable heterogeneity, which includes intertumor heterogeneity and intratumor heterogeneity (Wu et al., 2021). Intertumor heterogeneity mainly refers to the differences found between different malignant tumors and that of the same tumor among different patients. However, intratumor heterogeneity refers to the diversity of gene mutation spectrum and biological characteristics between tumor cells in different parts of the patient and in homogeneous tumors (Kalasekar et al., 2021). Tumor heterogeneity results from internal and external factors. Internal factors mainly include genomic instability and epigenetic variation (Bao et al., 2021). Among them, genetic instability (including mutation, chromosome instability, etc.) is the main internal cause of tumor heterogeneity. Additionally, epigenetic variation is another important internal factor of heterogeneity, which refers to the modifications of genes without changing the DNA sequence. This regulatory mechanism mainly includes DNA modification, chromatin accessibility, or gene expression regulation at the post-transcriptional level, which can affect gene expression and may lead to different phenotypes of tumor cells (Carter and Zhao, 2021; Haffner et al., 2021). The evolution of heterogeneous tumors conforms to the branch evolution model, that is, tumor cells obtain different mutations in the process of tumor development. In the theory of branching evolution, genomic instability leads to different genetic changes and different growth potentials of tumor cells. Epigenetic regulates the expression of tumor genes and forms different phenotypes. Both promote the tumor heterogeneity. However, external factors are mainly related to the tumor microenvironment (TME) (Bao et al., 2021). TME is mainly composed of tumor cells, immune cells, and stromal cells. Immune cells in innate and adaptive immune systems infiltrate into the TME, play the function of immune surveillance, and regulate the progress of tumor. Innate immune cells in solid tumors mainly include neutrophils, macrophages, dendritic cells (DC), mast cells, natural killer cells (NK cells), and myeloid inhibitory cells (MDSC), while adaptive immune cells mainly include T and B cells. Moreover, some nontumor-stromal cells (endothelial cells, fibroblasts, pericytes, and mesenchymal cells) are associated with these immune cells (Figure 1). All these cells and their secreted factors and molecules constitute the TME, which influences the drug resistance, immune escape, and metastasis of tumors (Chen et al., 2021).

In clinical practice, the treatment of cancer mainly includes surgery, radiotherapy, chemotherapy, targeted therapy, and immunotherapy. Unfortunately, treatment failure often occurs because drug resistance exists in all cancer therapeutic modes. The biological determinants of drug resistance include tumor growth kinetics, tumor heterogeneity, physical barriers, immune system, TME, and undruggable genome (Khatoon et al., 2020). Among these determinants, tumor heterogeneity is the main cause (Dagogo-Jack and Shaw, 2018). Tumor heterogeneity is normally reflected in its development, proliferation, invasion ability, and drug sensitivity (Wu et al., 2021; Bao et al., 2021; Kalasekar et al., 2021), thereby resulting in low therapeutic efficacy and high tumor recurrence rates. Studies have confirmed that the generation of acquired drug resistance is a direct consequence of primary tumor heterogeneity. Tumor heterogeneity either directly affects therapeutic targets or shapes the TME by defining transcriptomic and phenotypic profiles to influence drug resistance (Lawson et al., 2018; Marusyk et al., 2020; Hou et al., 2017) to modulate progression and therapeutic responses of tumor (McGranahan and Swanton, 2017).

Cancer immunotherapy is the treatment of cancer patients by stimulating the immune system to fight the disease (Van den Bulk et al., 2018). In addition to injecting cytokines and antibodies, directly inputting activated immune cells is also an important means of immunotherapy, known as immune cell therapy (Mizukoshi and Kaneko, 2019). There are at least five types of immune cell therapies so far, including LAK (lymphokine activated killer cells), TIL (tumor-infiltrating lymphocytes), CIK (cytokine-induced killer cells), DC-CIK (dendritic cell-cytokine induced killers), CAR-T (chimeric antigen receptor T cell) (Weber et al., 2020). However, immune cell therapy faces many challenges in solid tumors, including addressing tumor antigen heterogeneity, stromal disorders, and tumor accessibility, thereby managing toxicity and side effects (Cable et al., 2021). Moreover, the interdependent cellular composition, proportion, location, and motility in the TME demonstrate important effects on cancer immunotherapy efficacy (Pinato et al., 2020). Therefore, visualization of anti-tumor cells in the TME and exploring their interactions may help us identify patients who can benefit from treatments via better assessment of tumor heterogeneity, which is related to drug resistance and immune escape.

## Methods for Tumor Heterogeneity Dissection

Traditional methods for studying tumor heterogeneity mainly include immunofluorescence staining and flow cytometry (FCM) (Aldag et al., 2021). However, several major limitations of these methods exist, such as laborious and time-consuming processes, as well as low throughput in terms of genes that can be analyzed simultaneously, thereby holding back their wide applications in tumor heterogeneity study. Additionally, before the development of single-cell sequencing, a mathematical modeling method is often used for reconstructing tumor heterogeneity based on bulk sequencing, which is called tumor deconvolution (Schwartz Schäffer, 2017). Single-cell RNA sequencing (scRNA-seq) technology is an ideal tool for deciphering the heterogeneity in cell population. Compared to the conventional bulk sequencing approaches, single-cell sequencing possesses advantages for cancer heterogeneity studies in several aspects. First, scRNA-seq can help analyze the heterogeneity previously hidden in cell populations (Lei et al., 2021). Its application in tumor heterogeneity includes identifying rare or undefined cell types in tumors (Wagner et al., 2019; Wu et al., 2021) and classifying cell clusters according to their gene expression profiles (Ma et al., 2019). Second, scRNA-seq can also be applied to study the development trajectory of the classified clusters and determine the source of tumor cells (Zheng et al., 2017; Hu et al., 2021). Additionally, the mechanism of tumor heterogeneity and drug resistance can also be analyzed through the analysis of single-cell data (Sathe et al., 2020; Wang et al., 2021). Although scRNA-seq helps researchers better identify tumor cell subpopulations, this technology requires tissue digestion to release cells from their natural niches, thereby missing the original location coordinates of those cells. Nevertheless, the state of cancer cells is highly dependent on the precise spatial location and their interactions with adjacent cells. The lack of original location information of cells makes it difficult for scRNA-seq to study the functional interactions among different tissue regions. Thus, methods that can preserve the localization information of expressed genes have been developed and are recognized as spatial transcriptomic technologies (Asp et al., 2020).

## Spatial Transcriptomic Technologies

Visualizing gene expression *in situ* at the transcriptomic level can be achieved by RNA *in situ* hybridization (ISH) (Chu et al., 2019). However, conventional methods based on riboprobes often lack specificity and sensitivity. Also, the number of targets that can be analyzed simultaneously using conventional RNA ISH is limited by spectrally distinct labels. The RNAscope technology exhibits a unique probe design strategy, which allows signal amplification and background inhibition at the same time. It can realize single-molecule visualization and retain tissue morphology (Wang et al., 2012). The laser micro-dissection (LCM) technique is an early technology that can achieve high-throughput gene expression profiling *in situ*. LCM directly captures cell populations of interest from a complex heterogeneous tissue under a microscope (Espina et al., 2006). Thus, the captured cells can be subjected to various analytical procedures, e.g., RNA sequencing (RNA-seq) that exhibits high coverage and is capable of detecting almost all expressed genes in the region-of-interest of a sample. However, the cumbersome experimental processes and low-throughput hinder LCM to become a widely used spatial transcriptomic technology. Currently, mainstream spatial transcriptomic technologies are generally divided into two categories: the next-generation sequencing (NGS)–based approaches that uses barcoded primers to encode the positional information into individual transcripts before next-generation sequencing and the imaging-based approaches consist of the *in situ* sequencing-based methods wherein transcripts are amplified and sequenced in the tissue and the *in situ* hybridization-based methods wherein imaging probes are sequentially hybridized in the tissue (Rao et al., 2021) (Lewis et al., 2021).

In the first approach, RNA molecules in their original positions are released and captured by reverse transcription primers immobilized on the solid phase to generate cDNAs that integrated positional barcodes from the primers (Veselinyová et al., 2021). The tissue was fixed, stained, imaged, and permeabilized. During the penetration process, mRNA molecules spread vertically onto the chip surface and poly(A) tail of mRNA hybridized with the ploy d(T) reverse transcription (RT) primers, followed by reverse transcription *in situ*. Then, the resultant position barcoded cDNA is sequenced so that the expressed genes can be mapped back to their original tissue context by decoding. This is the first technology that is named spatial transcriptomics (ST). In the original ST, the capture area consists of 1,040 spots with a diameter of 100 μm. However, after 10x Genomics acquired this technology, it has been upgraded to 4,992 spots, with a smaller diameter of 55 μm, thereby providing higher resolution. With its commercial name Visium, since then, this new ST technology has been widely used, especially in the field of tumor research (Nerurkar et al., 2020; Maniatis et al., 2021). To provide an even higher spatial resolution, Chen et al. developed Slide-seq technology, which exploited unique DNA barcode encoded microbeads so that 10 μm resolution can be achieved in this way (Rodriques et al., 2019). However, these beads are deposited on the slide surface in a random manner such that their barcodes are required to be decoded by sequencing first to link their spatial location information with the captured RNAs. The decoding procedure may hinder its production efficiency and increase the costs.

The imaging-based spatial transcriptomic approaches can be divided into *in situ* sequencing-based methods and *in situ* hybridization-based methods. The first *in situ* sequencing (ISS) method was developed by Ke et al. in 2013, based on the original single-molecule RNA detection method using padlock probes and rolling circle amplification (RCA) (Ke et al., 2013; Larsson et al., 2010). In the original ISS, barcoded padlock probes were used to be ligated onto cDNAs generated from their target RNAs. After RCA for signal enhancement, barcodes are decoded using the sequencing-by-ligation chemistry. By linking the coordinate information from taken images and the decoded gene-specific barcodes, expressed RNAs are detected in their native tissue context. Several *in situ* sequencing methods have also been developed, such as fluorescent *in situ* sequencing (FISSEQ) (Lee et al., 2014), spatially-resolved transcript amplicon readout mapping (STARmap) (Wang et al., 2018), barcoded oligonucleotides ligated on RNA amplified for multiplexed and parallel *in situ* analyses (BOLORAMIS) (Liu et al., 2021), and expansion sequencing (ExSeq) (Alon et al., 2021). For *in situ* hybridization-based spatial transcriptomic methods, single-molecule fluorescence *in situ* hybridization (smFISH) can be regarded as their basis. Lubeck, et al. first showed that it is possible to perform sequential rounds of smFISH on the same RNA molecules, and different RNA species can be distinguished using the orders of dye labeling (Lubeck et al., 2014). Multiplexed error-robust fluorescence *in situ* hybridization (MERFISH) is another hybridization-based spatial transcriptomic method that is based on smFISH (Hospital et al., 2015). By sequential imaging using combinatorial probe sets as well as exploiting hamming code error correction in the barcoding strategy, MERFISH is able to detect thousands of genes at the same time. However, seqFISH+ achieved detection of 10,000 genes simultaneously using a similar probe design as in MERFISH (Chee-Huat et al., 2019), thereby achieving transcriptome-level gene expression profiling.

**FIGURE 2:**
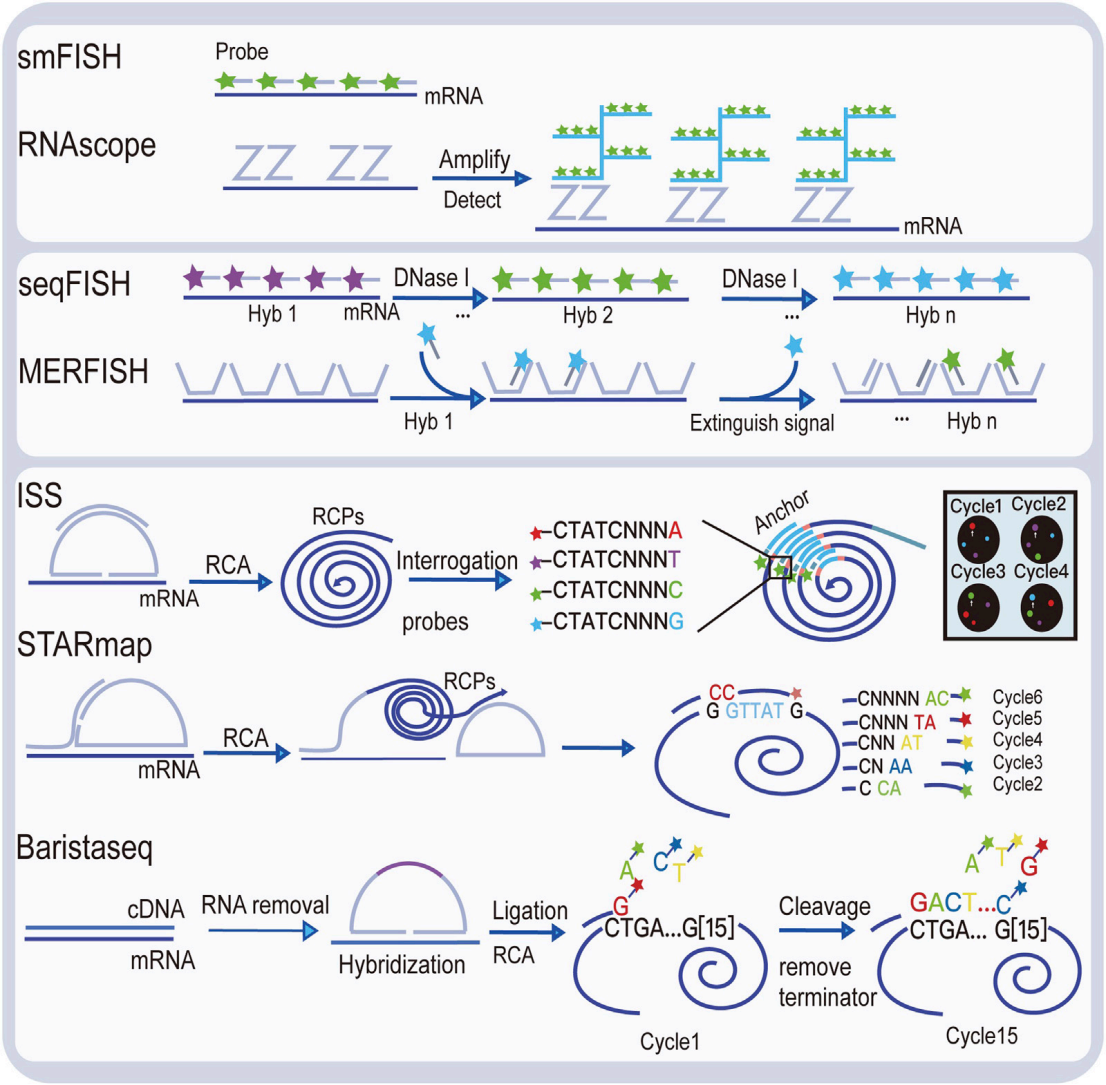
Illustration of image-based spatial transcriptomic technologies.

**FIGURE 3:**
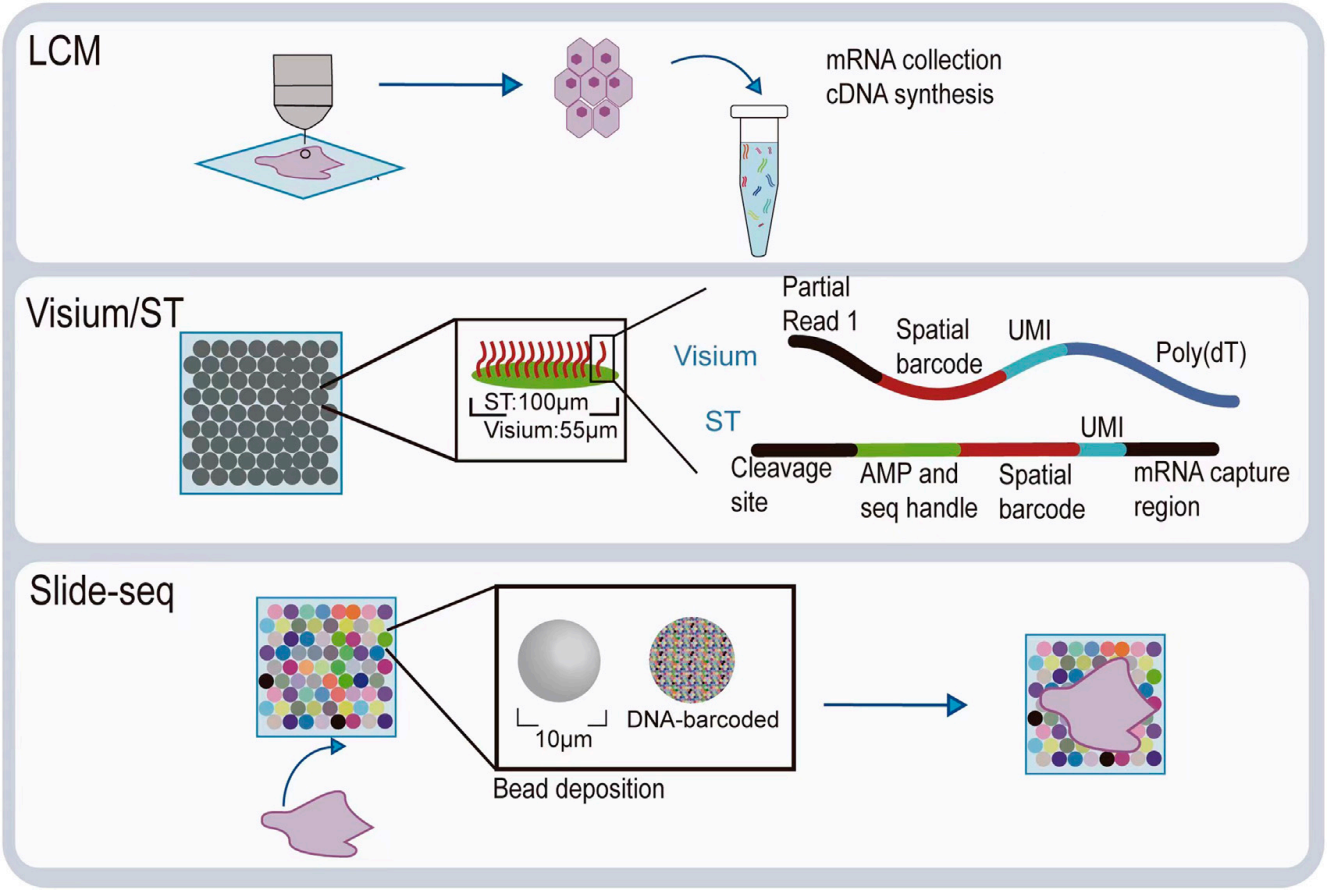
Illustration of spatial transcriptomic technologies based on *in situ* capture and next-generation sequencing.

Diagrams to explain the principle of spatial transcriptome technologies based on imaging and capture are shown in Figure 2 and Figure 3. Their advantages and drawbacks are summarized and compared in Table 1. By acquiring gene expression profiles, these ST technologies can be used to analyze the cell composition and their distribution in the TME, based on which their spatial trajectories and interactions can also be constructed. Thus, they help us better understand the pathogenesis, prognosis prediction, treatment, and prevention of cancer.

**TABLE 1:** Advantages and disadvantages of spatial transcriptomic technologies.

Classification	Method	Advantage	Resolution	Limitations	References
Methods based on imaging	Sm FISH	High efficiency	Single cell/subcellular	RNA detected is limited	Lubeck et al. (2014)
		Fresh-frozen and FFPE		Signals are required to be non-overlapping	
	FISSEQ	Each genomic site will have multiple probes	Single cell/subcellular	Probes need to be designed in advance	Lee et al. (2014)
		Strong signal			
	Seq FISH	Overcome optical congestion	Single cell/subcellular	High multiple need high cost	Chee-Huat et al. (2019)
	MERFISH	High efficiency	Single cell/subcellular	RNA detected is limited	Hospital et al. (2015)
	ISS	Fresh-frozen & FFPE	Single cell/subcellular	Probes need to be designed in advance	Ke et al. (2013)
		Detecting small RNA fragments			
	STAR map	High efficiency	Single cell/subcellular	Low throughput	(Wang et al., 2018)
	Baristaseq	Low autofluorescence background	Close to single cell	Displayed only on cultured cells	(Chenxy et al., 2018)
Methods based on capture	LCM	Target selection	Single cell	Incomplete tissue and cell	Espina et al. (2006)
	Visium/ST	High throughput	1–10 cells	Single cell resolution cannot be achieved	Nerurkar et al. (2020)
		Higher sensitivity		Expensive	Maniatis et al. (2021)
		Shorter experimental cycle			
	Slide-seq	High throughput	Close to single cell	Low magnetic bead capture efficiency	Rodriques et al. (2019)

## *In Situ* Cell Typing by Spatial Transcriptomics

With ST, it is possible to visualize different types of cells within tissue sections based on the spatial gene expression profiles. To reveal heterogeneity, researchers from Australia’s Institute of Natural Genetics developed a bioinformatic method called SCSubtype. First, they studied the biological pathways driving intratumor transcriptional heterogeneity (ITTH) and focused on 574 ITTH-related genes. These genes were divided into seven gene modules (GMs) with different functional characteristics. Through SCSubtype, they could classify, group, and analyze the functional enrichment of cells in breast recurrent tissues and score cells based on GMs (Wu et al., 2021). Next, to identify the cellular composition and malignant epithelial cells of primary breast cancer, they used canonical and cluster markers. Among them, genes associated with tumor metastasis and tumor cell proliferation, such as SPP1, GSTA1, MAL2, and MGST1, were highly expressed in LUSC, and significant enrichment of pathways associated with epithelial–stromal transformation was observed in the LUSC of spatial TME, thereby suggesting that LUSC exhibited a stronger invasive ability to transfer directly to the surrounding lymph nodes (Wu et al., 2021). The cellular composition and structure of human squamous skin cancer and normal skin were identified by single-cell sequencing combined with spatial transcriptome and multiplexed ion beam imaging by Ji et al. Cells were clustered into seven groups, including epidermal cells, fibroblasts, melanocytes, endothelial cells, NK/T cells, B cells, plasma cells, and myeloid cells. A specific subpopulation is found (MMP10^+^ and PTHLH^+^) in normal skin and epithelial cells, called the tumor-specific keratinocytes (TSK)-specific subgroup. Spatial transcriptomic results indicate that TSKs, basal tumor cells, and the fibrovascular niche adjacent to TSK constitute the heterogeneity at the leading edge of human skin squamous cell carcinoma (Ji et al., 2020).

## *In Situ* Gene Expression Profiling by Spatial Transcriptomics

Identifying spatially distinct gene expression patterns can reveal the biological processes in those regions. For example, Ståhl et al. identified six independent areas of pancreatic ductal carcinoma and invasive ductal carcinoma of breast cancer by ST (Ståhl et al., 2016). The results showed that the gene expression in the regions of ductal carcinoma demonstrated a surprisingly high heterogeneity. For example, the expression of KRT17 and GAS6 in epithelial mesenchymal transformation is particularly higher in regions 1 and 5. Hypoxia-related ENO1, LDHA, TPI1, ALDOA, MIF, and PGK1 were detected to be highly expressed in subgroup 6 of the hypoxia group. This study first describes the spatial gene expression changes of ductal adenocarcinoma of the breast induced by a hypoxic microenvironment, and it identifies its potential therapeutic targets, which can provide the foundation for further studies of prognosis and treatment of hypoxic tumors. Spatial heterogeneity of hypoxic tumors and control tissue in breast cancer was studied by Sun et al. (2021). ST results showed that tumor cell subgroups decreased to seven subsets compared to the nine subgroups of the normal control. Different subsets exhibit positional features and different gene features. Next, subgroups located at the forefront of invasion exhibited active functions under hypoxic conditions, including cell proliferation, invasion, and stress response, as well as the uneven distribution of hypoxia-related genes across subsets (Sun et al., 2021). These studies suggest that ST is able to dissect the heterogeneity in biopsy that cannot be detected by conventional transcriptome analysis, which may provide more detailed prognostic information. Results obtained by researchers from Australia’s Institute of Natural Genetics indicated that ST cluster analysis of recurrent breast cancer tissue samples yielded 10 clusters, of which LUAD and LUCS varied substantially in the spot types. Clusters were mapped back to spatially identified locations on their space and found to be consistent with anatomy. The genes associated with malignant tumors (SLPI, SCGB3A1, SCGB3A2, MS4A15, and NR4A1) were found to be highly expressed in LUAD1 tumor tissue. However, genes associated with tumor metastasis and tumor cell proliferation (SPP1, GSTA1, MAL2, and MGST1) were found to be highly expressed in LUSC1, LUSC2, LUSC4 samples. DEG function revealed the high activity of peptidase in the metabolic pathway in LUSC, thereby suggesting that the adhesion capacity between cell and substrate in the TME were different (Wu et al., 2021).

## Mapping Spatial Trajectory by Spatial Transcriptomics

Spatial transcriptomic technology can be used to map the spatial trajectory of tumors, such as metastasis and invasion. EMT refers to when epithelial cells are losing cell polarity and connectivity, adhesion, increase infiltration, migration, and become mesenchymal cells (Pastushenko and Blanpain, 2019). Zhang et al. collected lung tumor tissue from 12 individuals who received surgical resection (including four LUAD samples and eight LUSC samples) and performed spatial transcriptomic studies using Visium. The spatial trajectory analysis found that along the invasion trajectory of the tumor, the proportion of tumor spots gradually decreased, while interstitial spots increased. Tumor matrix contains immune macrophages, T cells, B cells, or other immune cell responses that affects the TME, thereby regulating tumorigenesis and development of tumor metastasis. Genes associated with tumor proliferation such as DSG2 and SPRR3 and genes related to energy metabolism process decreased along the invasion trajectory of the tumor, while the function of tumor progression-related gene BGN and metastasis growth-related gene POSTN gradually increased. Because tumor energy mainly comes from glycolysis and aerobic metabolism, and aerobic metabolism provides energy for tumor proliferation. The results showed that different lung cancer subclone cells were independent in their spatial distribution, with significantly different EMT between the subclones (Zhang et al., 2021).

## Discovery of Novel Spatially Dependent Mechanisms by Spatial Transcriptomics

Spatial transcriptomic technologies are expected to elucidate new mechanisms of spatial dependence in tumor pathogenesis. Sharma et al. analyzed 212,000 cells in human fetal, hepatocellular carcinoma (HCC), and mouse liver using scRNA-seq (Sharma et al., 2020). Combining with ST, they further revealed a common tumor–fetal ecosystem between fetal liver and HCC. Fetal-associated endothelial cells (PLVAP/VEGFR2) and fetal-like (FOLR2) tumor-associated macrophages were detected in stem cell carcinomas (Sharma et al., 2020). Furthermore, gene regulation analysis, ST, and functional analyses suggest important roles of VEGF and NOTCH signaling in maintaining the tumor–fetal ecosystem, thereby revealing a previously unexplored tumor–fetal reprogramming in the tumor ecosystem and providing new targets for therapeutic intervention in HCC (Sharma et al., 2020). New breakthroughs in the field of liver cancer have also been made using spatial transcriptomic technology. The human liver is the major site of tumor and metastasis. However, the molecular properties and cell–cell interactions of different cell types in liver pathologies are left to be explored. The mouse liver composition was studied using scRNA-seq and single-molecule RNA fluorescence *in situ* hybridization (smRNA-FISH). This study identified progenitor cells during the development and regeneration of liver, thereby describing the phenotypes of nonparenchymal cells in chronic liver disease and cirrhosis and revealing the heterogeneity of TME in HCC and a novel mechanism of the interaction between tumor epithelial cells and TME (Saviano et al., 2020). Next, single-cell RNA sequencing and spatial analysis of malignant and adjacent nonmalignant liver tissue from five patients with cholangiocarcinoma or liver metastases were performed by Massalha et al. (2020). This study found that stromal cells exhibited a recurrent and patient-independent expression programs. They reconstructed the ligand–receptor atlas, elucidated recurrent tumor–stromal interactions, provided resources for understanding human liver malignancies, and exposed potential intervention points (Massalha et al., 2020).

Moreover, the combined application of multi-omics also provides a reliable approach to discovering new mechanisms, which can also help in new target discovery as well as drug research and development. Ben-Moshe et al. used transcriptomics, miRNA array, and mass spectrometry proteome to reconstruct spatial maps of multiple regional features. They used banded surface markers to classify hepatocytes from high-resolution lobular regions of spatial resolution. The researchers found that some protein bands largely overlapped with the mRNA bands. These targets included the central peripheral Wnt receptors Fzd7 and Fzd8. Based on this result, they screened the periportal Wnt inhibitors targeting these receptors and found Ctnnbip1 to be a candidate (Ben-Moshe et al., 2019).

## Accessing Tumor Immune Microenvironment by Spatial Transcriptomics

The composition, proportion, location, and motility of the microenvironment, especially that of the immune cells, have important impacts on the progression and treatment response of cancer cells, and they are also one of the main determinants of patients’ prognoses (Lei et al., 2020). Therefore, to improve the efficacy of immunotherapy, spatial transcriptomic techniques can be used to identify sources of tumor heterogeneity and provide potential therapeutic targets for treatments. Ji et al. used RNA sequencing and ST to observe multiple features of potential immunosuppression in skin squamous cell carcinoma (cSCC), including the co-localization of T regulatory cells (Treg) with CD8 T cells in the separated tumor matrix. Also, the study defined the spatial niches of cSCC tumors and other cellular subsets and their communication gene network involved in cancer (Ji et al., 2020). Spatial transcriptomics was used to study the spatial gene expression in HER2-positive breast tumors. By integrating single-cell data, they spatially mapped tumor-associated cell types and found a tertiary lymphoid structure (TLS). TLS provides subtle microenvironments for anti-tumor and humoral immune system responses. This study confirmed the association of TLS prevalence with clinical outcome. Transcriptional analysis of TL-like structures is expected to reveal drug therapeutic effects and facilitate the study of immunity anticancer drugs (Andersson et al., 2021). Additionally, macrophages could be divided into LAM1 and LAM2 according to their status. In most cases, LAM1 and LAM2 cells show a negative correlation with each other, thereby suggesting that LAM cells could be polarized into LAM1 or LAM2 under different microenvironments. The Visium results produced by Swarbrick et al. indicated that LAM1 and LAM2 cells were present in the invasive cancer area and LAM2 cells were positively associated with CD4^+^ and CD8^+^ T cells. Next, genes co-expressed by both cells suggest that these cells demonstrate a functional immune regulation in some aspects (Wu et al., 2021). Moreover, 10x Visium was used to analyze tumor-associated macrophages (TAM) in patient biopsies by Nerurkar et al., and TAM was found to be associated with a poor prognosis. By constructing a glioma spatial transcription map, they found that microglia played a dominant role in the tumor infiltration, whereas the vessel-derived TAM was enriched near the vessels. Thus, blood-derived TAM was negatively associated with low-grade glioma. This finding supports the view that macrophage ontogeny is essential for cell morphogenesis (Nerurkar et al., 2020). Spatial transcriptomics combined with single-cell sequencing was used to analyze the expression of immune-related cells and chemokines and receptors in the TME in human squamous cell carcinoma, such as the CXCR3 (Treg, CD8^+^TEM), CXCR6 (Treg, CD4^+^RGCC^+^, and CD8^+^ depleted cells), and CXCR4 (CD4^+^RGCC^+^, CD8^+^ TEMRA, and NK cells). CCR8 is specifically expressed in Treg, thereby suggesting that it is a potential therapeutic target to inhibit Treg recruitment. Also, the results showed that CD8^+^ T cells, Treg, and macrophages were highly correlated with CD4^+^ T cells. Besides, fibroblasts, macrophages, and Treg were most abundant at the tumor–stromal margin, while most CD8^+^ T cells and neutrophils were excluded from the tumor, thereby suggesting that Treg prevents effector lymphocytes from entering the tumor (Ji et al., 2020). These results further reveal multiple immunosuppressive cell types and their involvement in the human squamous skin cancer microenvironment.

## Dissecting Tumor Matrix Microenvironment by Spatial Transcriptomics

TME is closely related to tumor drug resistance. Tumor-related macrophages (TAM), tumor-related fibroblasts (CAF), and tumor-related MSC (TA-MSC) in TME can enhance tumor resistance by recruitment and secretion of a variety of protective cytokines (Xu et al., 2021). Noncellular components, such as extracellular matrix, hypoxia, and acidification, can mediate drug resistance by constructing physical barriers and affecting the growth and metabolism of tumor cells (Bai et al., 2018). ECM (Extracellular Matrix), as a physical barrier of tumors, can dissolve or delay the delivery of drugs and ECM remodeling that cause tumor cells to escape apoptosis, cancer stem cell heterogeneity, and tissue polarity. Also, ECM can promote tumor resistance by activating survival-related pathways. The growth of malignant cells is driven by interactions between tumor cells and the stromal cells that constitute the TME (Marozzi et al., 2021). Although the role of mesenchymal cells in anti-tumor immunity has been demonstrated, the interaction between immune and stromal cells has not been clearly elucidated (Li et al., 2021).

To further understand the heterogeneity of CAFs in the TME, breast cancer matrix-enriched samples of 4T1 mice were analyzed using scRNA-seq and ST by Grauel et al. They analyzed the composition of tumor-associated fibroblasts (CAFs) and found the presence of four subpopulations in CAFs. Each subgroup demonstrates a distinct phenotype and function. Subset 1 (inflammatory CAFs, iCAFs) was significantly enriched in inflammatory and immune-related pathways, and subset 2 (typical myofibroblasts, myCAFs) was significantly associated with ECM deposition. This indicates that these cells play an important role in the matrix composition of the tumor framework. Subset 3 (VEGF^+^ CAFs), characterized by glycolysis and carbon metabolism, were found to be enriched in pathways associated with metabolic regulation. Next, subset 4 (proliferative type CAFs, prCAFs) is dominated by cell cycle–related features and expresses many genes associated with cell cycle progression (Grauel et al., 2020). Also, the subclass of CAF was demonstrated using the deconvolution algorithm by Swarbrick et al. They found that CAF like myofibroblast was enriched in the invasive cancer area, while ICAF are distributed in the aggregation area of invasive cancer, stroma, and lymph nodes. Meanwhile, in localization analysis of different cells, ICAFs were also found to be associated with CD4+/CD8+T cells, thereby indicating that they were related to invasive breast cancer with high TIL infiltration or immunological inflammatory phenotype (Wu et al., 2021).

ST combined with scRNA-seq can also be used to draw a new heterogeneous cell communication map to analyze the mechanism of stromal and immune cells in TME. The interaction of the ICAF ligand receptor and CD4^+^/CD8^+^ T cells was studied by Swarbrick et al. They found that immunomodulatory ICAF ligand and homologous T cell receptor, including chemokine (CXCL12/CXCL14-CXCR4 and CXCL10-CXCR3), supplementary pathway, transforming growth factor-β (TGFB1/TGFB3-TGFBR2), and lymphocyte inhibitory/activating molecules (LTB-LTBR, TNFSF14-LTBR and LTB-CD40, and VTCN1/B7H4-BTLA) were distributed in adjacent areas, thereby further revealing the regulatory effect of CAFs on immune cells (Wu et al., 2021). In the field of pancreatic ductal carcinoma, the multimodal intersection analysis (MIA) method was developed by Moncada et al. to integrate scRNA-seq and ST data. A small cohort of six patient samples (10 slices) was analyzed by MIA to map the status of cancer cells in different spatial tissue regions and to describe the interaction between cancer cell subsets and other cell subsets. The results indicated that spots with high expression of the stress module gene were significantly correlated with inflammatory fibroblasts (Moncada et al., 2020). Next, two new cell subsets of pancreatic ductal carcinoma (hypoxic ductal cells and antigen-presenting ductal cells) were also found in the study (Moncada et al., 2020).

Additionally, other stromal cells, such as TSK populations, are also involved in cellular communications in the TME. A combined analysis of ST and single-cell sequencing can reveal the communication network among cells. By using this approach, TSK was found to be located in the fibrovascular niche. Further, integrating single-cell and spatial data and mapping ligand–receptor networks to specific cell types can reveal that TSK cells are the hub of intercellular communication (Ji et al., 2020).

## Novel Pathological Classification by Spatial Transcriptomics

Distinguishing ductal carcinoma *in situ* (DCIS) from invasive ductal carcinoma (IDC) region biopsies is a clinical diagnostic challenge. ST can quantify and visualize the transcriptomes in tissue sections, and they can identify different pathological classifications of breast cancer (nonmalignant, DCIS, and IDC) after machine learning. Four published ST datasets for breast cancer were used to build machine learning models to determine the expression characteristics of different pathological regions (nonmalignant, DCIS, and IDC). According to the automatic recognition and expression feature classification of all ST spots that covered tissue sections, the prediction accuracy of DCIS and IDC was 95 and 91%, respectively (Yoosuf et al., 2020). This study suggests that ST technology is expected to provide clinical decision support for pathologists in the future. Classification based on spatial transcriptomic gene features in lung cancer (Zhang et al., 2021) and breast cancer (Wu et al., 2021) is also consistent with the traditional pathological classification.

Additionally, pathological classification based on ST can obtain histological classification information that is not available using traditional pathology. ST was used to explore the transcriptomes of nearly 6,750 tissue spots and determine the expression profiles of different tissue components (e.g., matrix, normal and needle glands, immune cells, and cancer) by Berglund et al. This study found that this gene-based classification method provides a more accurate description of the range of cancer foci than the pathologist’s annotation (Berglund et al., 2018). Additionally, the ISS method was used to spatially resolve the expression of ER (ESR1), PR (PGR)HER2 (ERBB2), KRT5/6/8, KI67 (MKI67), and EGFR within breast cancer tissue sections covering luminal A/B-like, HER2-positive, and triple negative tumors by Nilsson et al. These genes were used to approximate the breast cancer molecular subgroups according to the following criteria: luminal A-like (ER+ and/or PR+, HER2-, KI67 low, and KRT8+), luminal B-like (ER+ and/or PR+, HER2-, KI67 high, and KRT8+), HER2-positive (ER−/+, PR−/+, and HER2+), and triple negative breast cancer (TNBC) (ER-, PR-, HER2-, EGFR+, and/or KRT5/6+). Of note, gene expression based on total ISS read counts correlated with the microarray data for all subtyping genes except for PR (Svedlund et al., 2019). However, when comparing the microarray data with the spatially-resolved ISS data and the immunohistochemical results, three tumors that were negative for PR with microarray data were positive with ISS and in protein staining. Discrepancies were observed for two tumors positive for PR with ISS (in 81% and 31% of tumors, respectively) but negative with microarray and histological protein staining. PR expression was in agreement with the ISS data and indicated that tumor tissues were highly heterogeneous in their distribution of PR-positive cells, which could explain the discrepancies in the microarray and protein data. In the study of cutaneous malignant melanomas, similar findings have been found (Berglund et al., 2018). ST yield many pathologically unavailable information, which is expected to be linked to clinical features. A deep learning model was established by Jurgenson et al., which is able to spatially resolve the large number of mRNA and miRNA expression levels on pathological whole slide images (WSIs). They applied this method to breast and lung cancer slides and produced tumor WSI heterogeneity maps and calculated the heterogeneity index (HTI). This strategy promises to open up a new and viable avenue for studying tumor heterogeneity and other spatial molecular properties as well as their association with clinical features, including therapeutic sensitivity and survival (Thrane et al., 2018). Additionally, obtaining metastasis information of cancer is critical for clinical treatment. Accurate determination of migration patterns from somatic mutation data is complicated by intratumor heterogeneity and discordance between clonal lineage and cellular migration. A new algorithm called “Metastatic and Clonal History Integrative Analysis” was developed by Dr. Ben Raphael’s Lab of Princeton University. This algorithm can track the spread of cancer cells from one part of the body to another by combining the DNA sequence data with the information on cells in the body. Understanding the drivers of metastasis may be helpful for new treatments to prevent the spread of cancer in the body. Raphael and his team applied this method to breast cancer data and analyzed metastasis patterns in patients with melanoma, ovarian cancer, and prostate cancer. Raphael also plans to make this algorithm more effective by combining the data of tumor DNA and tumor cells circulating in the blood, as well as the epigenetic changes of DNA-reversible chemical modification (El-Kebir et al., 2018).

## Prediction of Disease Prognosis by Spatial Transcriptomics

The ecotype of a tumor is related to the survival and prognosis of patients. Identification of ecotypes of tumor patients by ST can be used to predict the prognosis of tumor patients. A deconvolution analysis of the spatial transcriptomic dataset of primary breast tumor data by Swarbrick et al. revealed a large number of circulating cells distributed in the basal base, LUMB, and HER2 enrich (HER2E) tumor tissues. Consensus cluster divides the breast cancer cohort into nine tumor clusters with similar cellular components, called ecotypes. These ecotypes show correlations with tumor subtypes, SC subtype cell distribution, and the diversity of major cell types. Ecotype 4 (E4) is a highly enriched immune cell associated with anti-tumor immunity, including depleted CD8^+^T cells, TH1, and central memory CD4^+^T cells. Ecotype 2 (E2) mainly includes LUMA and normal tumor cells, as well as mesenchymal cells, including endothelial CXCL12^+^ and ACKR1^+^ cells, s1-MSC ICAF, and exhausted circulating cells. Among these, the prognosis of patients with E2 tumor is best and that of E7 is poor, which may be related to the enrichment of Her2E cells (Wu et al., 2021). The ISS-based molecular subtyping and OncotypeDX recurrence score were established by Nilsson et al. The OncotypeDX recurrence score includes the expression of 21 genes divided into four functional groups, namely, HER2, ER, proliferation, and invasion. To spatially resolve the expression of a gene related to OncotypeDX recurrence score, they used the ISS method. Based on the published algorithm for ISS data, each gene group was scored, and the recurrence score was then calculated. Six of the tumors displayed high recurrence score, one tumor showed a moderate score, and two tumors displayed a low recurrence score. The OncotypeDX score based on total ISS read counts was consistent with previous scores based on RT-qPCR performed on the same tumors (Svedlund et al., 2019). These results indicate that the ST technology was well suited for tumor subtyping and prognosis evaluation. Liver metastasis, the leading cause of colorectal death, exhibits high immune microenvironment heterogeneity and inhibition. Furthermore, spatial transcriptomics analysis of tumor immunophenotype can predict the prognosis of tumor patients. 10x Genomics single cell sequencing combined Visium ST and multiplex immunofluorescence was used to deeply analyze the immunodynamic changes of liver metastasis in colorectal cancer by Gao and Zhang. The researchers identified myeloid cells, CD8^+^ T cells, CD4^+^ T cells, NK cells, and B cells from liver metastasis samples of colorectal cancer. Immunophenotype was found to undergo anti-tumor remodeling after neoadjuvant chemotherapy treatment in responsive patients. However, nonresponsive patients exhibit more immunosuppression, which is related to their *in vivo* immunosuppressive cells, such as MRC1^+^CCL18^+^M2-like macrophages, being reprogrammed at metastatic sites (Wu et al., 2022). This study describes the immune evolution process of metastasis and reveals how tumors respond to neoadjuvant chemotherapy. This is a successful case of ST combined with single-cell sequencing in clinical efficacy evaluation and prognostic prediction.

## Conclusion

Intratumor heterogeneity poses significant challenges to accurate diagnosis and personalized treatment of cancer. ST has been widely used in the field of tumor heterogeneity research, and its combination with scRNA-seq analysis provides new analytical dimensions for the cell subtype identification, spatial distribution, and movement trajectory of cells in tumor tissues. Additionally, the construction of spatial transcriptomics map improves understanding of the TME. Besides, studies combining spatial transcriptomes with mass spectrometry (Ali et al., 2020; Jackson et al., 2020; Goltsev et al., 2018) or high dimensional immunofluorescence (Saka et al., 2019; Lin et al., 2018; Schwartz et al., 2020) methods will help explore the extent and origins of tumor heterogeneity and obtain information for targeted diagnosis and treatment. Proteomics based on mass spectrometry and metabolomics technology can effectively determine the differences in protein (modified protein) or metabolism of different heterogeneous tumors by simultaneously analyzing the protein expression, post-translational modification level, and metabolite level in tumor cells, tissues, or body fluids with different heterogeneous phenotypes. Further, it can provide a powerful tool to further explain the molecular mechanism of heterogeneity and explore intervention pathways.

Although possessing great potential for studying tumor heterogeneity, current spatial transcriptomic technologies also face many challenges. Technically, relatively high costs and laborious experimental procedures, limited area of tissue that can be analyzed, and difficult to achieve three-dimensional gene expression profiling still remain problems for ST technologies to overcome. Scientifically, before it can be used in clinical settings, enough data to support the links of spatial gene expression patterns to the development, treatment, diagnosis, and prognosis still need to be accumulated and analyzed. Nonetheless, the combination of spatial multi-omics, including ST, will provide new ideas and insights into screening tumor drug targets, improving the accuracy of clinical diagnosis, and exploring new therapeutic approaches in the near future.
